# Cost-consciousness among Swiss doctors: a cross-sectional survey

**DOI:** 10.1186/1472-6963-5-72

**Published:** 2005-11-10

**Authors:** Patrick A Bovier, Diane P Martin, Thomas V Perneger

**Affiliations:** 1 Department of community medicine, Geneva University Hospitals, 24 Micheli-du-Crest, CH-1211 Geneva 14, Switzerland; 2 Quality of Care Unit, Geneva University Hospitals, 24 Micheli-du-Crest, CH-1211 Geneva 14, Switzerland; 3 Department of Health Services, Box 357660, University of Washington, Seattle, WA 98195, USA

## Abstract

**Background:**

Knowing what influences physicians attitudes toward health care costs is an important matter, because most health care expenditures are the results of doctors' decisions. Many decisions regarding medical tests and treatments are influenced by factors other than the expected benefit to the patient, including the doctor's demographic characteristics and concerns about cost and income.

**Methods:**

Doctors (n = 1184) in Geneva, Switzerland, answered questions about their cost-consciousness, practice patterns (medical specialty, public.vs. private sector, number of patients per week, time spent with a new patient), work satisfaction, and stress from uncertainty. General linear models were used to identify independent risk factors of higher cost-consciousness.

**Results:**

Most doctors agreed that trying to contain costs was their responsibility ("agree" or "totally agree": 90%) and that they should take a more prominent role in limiting the use of unnecessary tests (92%); most disagreed that doctors are too busy to worry about costs (69%) and that the cost of health care is only important if the patient has to pay for it out-of-pocket (88%). In multivariate analyses, cost-consciousness was higher among doctors in the public sector, those who saw fewer patients per week, who were most tolerant of uncertainty, and who were most satisfied with their work.

**Conclusion:**

Thus even in a setting with very high health care expenditures, doctors' stated cost-consciousness appeared to be generally high, even though it was not uniformly distributed among them.

## Background

Because most health care expenditures are the results of doctors' decisions, whether doctors are cost-conscious is an important matter. Many decisions regarding medical tests and treatments are influenced by factors other than the expected benefit to the patient, including the doctor's demographic characteristics [1,2], training [3-6], work context [7,8], financial incentives [9,10] and information about costs [11-13]. Medical decisions are also influenced by subjective considerations, including risk aversion [14,15], tolerance for uncertainty [16], and concerns about cost and income [17].

In this paper, we are interested in cost-consciousness, defined as a concern to contain costs of health care borne by society [18]. Cost-consciousness was the first factor identified from a series of different attitudes hypothesized to influence physicians' resource use [19], based on a conceptual model of medical care process [20] and a review of physician decision making [21]. Goold et al. found that cost-consciousness was associated with lower self-reported estimates of resource use. However, whether cost-consciousness leads to less costly medical care is not known. While those who pay for health care may regard this evolution as desirable, cost-consciousness creates a possible conflict of interest. According to traditional medical deontology, the sole concern of a doctor should be the welfare of the patient, regardless of costs to society [22]. The tensions caused by such conflicts of interest are particularly acute in managed care settings [23,24]. Thus doctors may have good reasons not to be cost-conscious.

The aims of this study were to assess the cost-consciousness of doctors practicing in public and private settings in the Swiss canton Geneva, and to identify practice patterns and doctors' characteristics associated with cost-consciousness.

## Methods

### Setting

Switzerland has the second highest health care expenditures per capita in the world, behind the United States, and devotes about 11% of the GNP to health care [25,26]. Doctors in private practice who are paid on a fee-for-service schedule provide most ambulatory care, and most hospitals are public, subsidised by local governments, where doctors are salaried (in addition, some senior hospital doctors have private practice privileges). To achieve social solidarity, the Health Insurance Law makes compulsory the purchase by households of a fairly comprehensive package of health benefits, which includes ambulatory treatment, inpatient care, home nursing care, and some health promotion activities. The basic health insurance coverage can be contracted from approximately 90 private insurance carriers, which are not allowed to earn profits from the mandated benefit package. Insurers can offer different schemes for health care provision such as patients' free choice of physician ("any-willing-provider" or compulsory contracting) or preferred providers' contracts (general practitioner-gatekeeper model or restricted network of providers). Restriction on the choice of the provider results in lower premiums for the patient. According to official statistics, in 2001, about 45% of the insured chose the lowest permissible deductible and only 9% the highest; about 8% chose a managed care policy, mainly the general practitioner-gatekeeper model. Regarding the organisation of care, the Swiss health care system has therefore been qualified as a "regulated competition system without managed care" [27]. The canton Geneva has the highest health care costs and medical density in the country. In 1998, there were 58 doctors per 10000 residents in canton Geneva – 31 in private practice, and 27 in public hospitals.

### Study design

We conducted a cross-sectional mail survey of doctors practicing in canton Geneva, Switzerland, during the fall of 1998. Doctors were identified from membership files of the Geneva Medical Association (1370 members) and the Swiss Association of Interns/Registrars, Geneva Section (906 members). After exclusion of 54 duplicate records, 10 pre-test participants, 97 doctors who had incorrect addresses and 121 who did not practice clinical medicine, 1994 doctors were eligible.

### Measurement of cost-consciousness

Attitude toward costs (cost-consciousness) was measured with a validated 6-item instrument [19]. Two items explore the doctors' opinion about health care costs in general, three items explore their attitudes regarding the costs of tests and procedures, and a last item probes the importance of out-of-pocket payments (Table 1). The items were scored on a five-point Likert scale anchored by 1: totally disagree and 5: totally agree. Negatively worded items were reversed so that a higher score would mean greater cost-consciousness. We translated the instrument into French using a standardized procedure (three independent translations, selection of a consensus translation by an expert panel, pre-tests among 10 physicians) (Appendix 1). The internal consistency of the translated scale was similar to that of the original scale (Cronbach α : 0.68, versus 0.74 for original) and a factor analysis indicated that the translated items represented a single dimension. We computed a summary cost-consciousness score whenever at least 3 of the 6 items were present.

**Table 1:** Original English and French translation of the cost-consciousness scale.

Trying to contain costs is the responsibility of every physician.	Essayer de maîtriser les coûts de la santé est la responsabilité de chaque médecin.
There is currently too much emphasis on costs of tests and procedures. *	Actuellement, on met trop l'accent sur le coût des examens et des interventions médicales. *
Doctors need to take a more prominent role in limiting use of unnecessary tests.	Les médecins doivent jouer un rôle plus important dans les efforts pour limiter l'emploi d'examens inutiles.
Doctors are too busy to worry about the costs of tests and procedures. *	Les médecins sont trop occupés pour se soucier du coût des examens et des interventions médicales. *
The cost of a test or medication is only important if the patient has to pay for it out-of-pocket. *	Le coût d'un examen ou d'un traitement est important seulement si le patient doit le payer de sa poche. *
It is unfair to ask physicians to be cost-conscious and still keep the welfare of their patients foremost in their minds. *	Il est injuste de demander aux médecins de penser à réduire les coûts de la santé, tout en se préoccupant avant tout du bien-être de leurs patients. *

Answer scale : 1 : Totally disagree/Pas du tout d'accord – 5 : Totally agree/Tout à fait d'accord *: reversed score for the summary scale

### Predictors of cost-consciousness

Determinants of cost-consciousness included socio-demographic characteristics (age, sex), time since graduation from medical school, type of practice (public versus private), medical specialty, workload characteristics (number of patients per week, time spent with a new patient), stress from uncertainty, and work-related satisfaction.

Stress from uncertainty refers to physicians' reaction to their limitations of professional knowledge, problems of diagnosis, ambiguities of treatment and outcome, unpredictability of patients response, and variations in physicians' attitudes, values and perceptions of risk [28-33]. Items of a stress from uncertainty scale [28] were translated into French using the same procedure as for the cost-consciousness scale. Factor analysis confirmed that the items represented a single dimension. The internal scale consistency coefficient was 0.88.

Work-related satisfaction was measured by a 17-item instrument based on the work of the Society of General Internal Medicine Career Satisfaction Study Group [34], containing questions addressing satisfaction with workload, intellectual stimulation, leisure time, work stress, relations with patients, autonomy in treating patients, autonomy in specialist referrals, overall quality of care, relations with peers, relations with nurses and other non medical staff, administrative burden, continuing medical education, enjoyment of work, respect and prestige, payment mechanism, current income, and job satisfaction overall [35]. The internal scale consistency coefficient of a work related satisfaction scale including all 17 items was 0.88.

### Data analysis

Descriptive statistics were calculated for each item of the cost-consciousness instrument (mean, standard deviation, floor & ceiling effect). Summary scores were calculated by averaging the responses to the relevant items, whenever half or more were present. For comparisons with the original [19], the cost-consciousness score was rescaled between 0 and 24; to facilitate the interpretation of regression models, we also standardized this score to mean 50 and standard deviation 10.

We explored the relationships of the cost-consciousness scale with socio-demographic and job characteristics of the respondents, stress from uncertainty, and work related satisfaction. We used analysis of variance, including tests for linear trend where indicated, to test these associations. Because units of all scales are arbitrary, we categorized the continuous predictors into quartiles, because correlation or regression coefficients can be difficult to interpret, while quartiles are more intuitive. Furthermore, this approach also allows the reader to check whether the association is linear or not. Multivariate models were used to adjust for potential confounders, identify independent risk factor of higher cost-consciousness score, and compute adjusted means. All statistical tests were two-tailed, with a significance level of 0.05.

## Results

After the first mailing and two reminders, 1184 doctors (59%) responded to the survey. Two thirds were men (784, 66%) and their mean age was 44.9 years (SD: 10.9). Most respondents were in private practice (748, 63%), 362 (31%) were hospital interns or registrars in the public sector, and 67 (6%) held senior posts in the public hospitals (7 missing data). Respondents included 402 primary care doctors (generalists, general internists: 34%), 196 internal medicine specialists (17%), 83 paediatricians (7%), 178 psychiatrists (15%), and 325 (27%) other specialists (surgical specialties, radiologists). On average, they had graduated from medical school 17.8 years earlier (SD: 10.3).

### Doctors' attitude toward health care costs

The distribution of the answers to most items was skewed toward the higher end of the response scale (Table 2). Almost all doctors (90%) agreed that trying to contain costs was their responsibility, and that they should take a more prominent role in limiting the use of unnecessary tests (92%). A majority of respondents (69%) disagreed with statements that doctors are too busy to worry about costs, and that the cost of a medication is only important if the patient has to pay for it out-of-pocket (88%). Only one out of four doctors (27%) thought that it is unfair to ask doctors to be cost-conscious and still keep the welfare of their patients foremost in their minds. Almost two-thirds (61%) agreed that there is currently too much emphasis on the costs of tests and procedures. The cost-consciousness score could be computed for 1176 respondents (99%); its mean was 16.8, SD 3.6, and quartiles 13.8, 16.8, and 19.2 when scaled between 0 and 24 (21). Its ditribution was close to normal.

**Table 2:** Descriptive statistics of the items of the cost-consciousness scale of 1184 doctors in Geneva, Switzerland, 1998.

	Percent responding						
		
Item	Missing	Totally disagree	Disagree	Not sure	Agree	Totally agree	Mean (SD)
Trying to contain costs is the responsibility of every doctor.	0.6	1.5	2.7	5.8	45.8	44.2	4.3 (0.8)
There is currently too much emphasis on costs of tests and procedures. ^a^	1.4	5.2	18.4	15.8	42.2	18.3	2.5 (1.1)
Doctors need to take a more prominent role in limiting use of unnecessary tests.	0.9	0.7	1.4	6.2	50.6	41.1	4.3 (0.7)
Doctors are too busy to worry about the costs of tests and procedures. ^a^	0.8	33.6	35.0	16.2	12.3	2.9	3.8 (1.1)
The cost of a test or medication is only important if the patient has to pay for it out-of-pocket. ^a^	0.9	59.7	28.7	6.6	3.8	1.3	4.4 (0.9)
It is unfair to ask doctors to be cost-conscious and still keep the welfare of their patients foremost in their minds. ^a^	1.4	19.6	34.7	18.8	19.0	8.0	3.4 (1.2)

^a^: Negatively worded items were reversed so that a higher score would mean greater cost-consciousness.

### Relationship to socio-demographic and work-related characteristics

Cost-consciousness was similar in men and women (Table 3), but younger doctors had slightly higher scores. Doctors in the public sector were in general more cost-conscious, in particular the senior staff across all age categories (results not shown). Internal medicine specialists and paediatricians had higher cost-consciousness scores, while doctors in surgical and technical specialties had the lowest (results not shown). Doctors who saw fewer patients per week and spend more time with each new patient were also more cost-conscious. Cost-consciousness scores were also higher for doctors who reported lower stress from uncertainty and higher work-related satisfaction (Table 3).

**Table 3:** Relationships of cost-consciousness to socio-demographic and work-related characteristics of 1184 Swiss doctors, Geneva in Switzerland, 1998.

	N	*%*	Cost-consciousness T score	P value
Sex				*0.68*^*a*^
Men	784	*66*	50.1	
Women	400	*34*	49.8	
Age (years) (21 missing)				*0.05*^*a*^
<35	263	*23*	50.4	
35–50	568	*49*	50.5	
>50	332	*28*	48.9	
Years since graduation from medical school (17 missing)				*0.22*^*a*^
0–10	325	*28*	50.5	
11–17	290	*25*	49.7	
18–24	272	*23*	50.6	
>24	280	*24*	49.1	
Type of practice				*0.001*^*a*^
Public sector, in training	368	*31*	50.9	
Public sector, senior staff	68	*6*	53.5	
Private sector	748	*63*	49.3	
Self-reported number of patients per week (83 missing)				*<0.001*^*b*^
<26	269	*24*	51.6	
26–50	409	*37*	50.1	
51–75	195	*18*	49.1	
>75	228	*21*	48.6	
Self-reported time spent (minutes) with a new patient (66 missing)				*0.001*^*b*^
<31	382	*34*	48.6	
31–45	324	*29*	50.2	
46–60	344	*31*	50.4	
>60	68	*6*	52.5	
Stress from uncertainty (20 missing)				*<0.001*^*b*^
Lowest quartile (1 – 2.13)	269	*23*	51.8	
2^nd ^quartile (2.15 – 2.67)	305	*26*	50.6	
3^rd ^quartile (2.69 – 3.23)	336	*29*	50.3	
Highest quartile (3.25 – 5.0)	254	*22*	46.9	
Work-related satisfaction (9 missing)				*<0.001*^*b*^
Lowest quartile (1.0 – 4.5)	284	*24*	48.2	
2^nd ^quartile (4.53 – 5.0)	338	*29*	49.6	
3^rd ^quartile (5.06 – 5.47)	271	*23*	50.7	
Highest quartile (5.5 – 7.0)	282	*24*	51.6	

^a^: ANOVA, difference between groups

^b^: ANOVA, test for linearity.

### Multivariate analyses

Multivariate analysis identified the following independent risk factors for higher cost-consciousness: type of practice (doctors in public sectors had higher scores), lower workload (number of patients per week), lower stress from uncertainty, and higher work-related satisfaction (Table 4). Other factors that were significant in the univariate analyses, such as specialty and self-reported time with a new patient, were not anymore significant after adjustment for type of practice, self-reported number of patient seen per week, stress from uncertainty and work related satisfaction. No sociodemographic characteristics were statistically associated with cost-consciousness in the multivariate analysis. We also explored the possible interactions between these factors, but none were significant.

**Table 4:** Multivariate predictors of cost-consciousness in 1184 Swiss doctors, Geneva, Switzerland, 1998.

	Adjusted mean cost consciousness	95% CI	P value
Type of practice			*0.005*^*a*^
Public sector, in training	51.2	50.0 to 52.4	
Public sector, senior staff	51.8	49.3 to 54.3	
Private sector	49.0	48.2 to 49.8	
Self-reported number of patients per week			*0.002*^*b*^
<26	51.9	50.6 to 53.2	
26–50	51.2	50.0 to 52.4	
51–75	50.3	48.7 to 52.0	
>75	49.1	47.5 to 50.7	
Stress from uncertainty			*<0.001*^*b*^
Lowest quartile (1 – 2.13)	52.4	51.0 to 53.9	
2^nd ^quartile (2.15 – 2.67)	51.4	50.1 to 52.8	
3^rd ^quartile (2.69 – 3.23)	50.9	49.5 to 52.2	
Highest quartile (3.25 – 5.0)	47.9	46.4 to 49.4	
Work-related satisfaction			*<0.001*^*b*^
Lowest quartile (1.0 – 4.5)	48.5	47.0 to 49.9	
2^nd ^quartile (4.53 – 5.0)	50.3	48.9 to 51.6	
3^rd ^quartile (5.06 – 5.47)	51.3	49.9 to 52.8	
Highest quartile (5.5 – 7.0)	52.5	51.0 to 54.0	

^a^: ANOVA, difference between groups

^b^: ANOVA, test for linearity.

## Discussion

In a setting where medical density and health care expenditures are particularly high, we found that a majority of doctors agreed that trying to contain costs was their responsibility, that they should worry about the costs of tests and procedures they order, that they should take a more prominent role in limiting the use of unnecessary tests, and that the cost of a test or medication is not only important if the patient has to pay for it out-of-pocket. In multivariate analyses, cost-consciousness was higher among doctors in the public sector, those who saw fewer patients a week, those who were most tolerant of uncertainty, and those most satisfied with their work.

The absolute levels of cost consciousness were similar in our sample and in the previous report from an academic medical centre in the United States [19]: on a scale of 0 to 24, the mean was 16.8 (SD 3.6) in Geneva, Switzerland, and 17.2 (SD 3.1) in Ann Arbor, Michigan. Whether attitudes toward cost-containment are different in other settings would require other similar surveys.

### Practice patterns

The difference between the private and the public sectors may have several origins. It is possible that working in a public hospital raises the doctors' sensitivity to health care costs. Alternatively, the public sector may attract doctors who are more cost-conscious than those who choose the private sector. This issue could be resolved by following doctors prospectively, as they move from the public hospital into private practice.

Regardless of the setting, doctors who saw fewer patients per week were also more cost-conscious. In a fee-for-service system, a doctor's income is directly related to the number of patients seen. Doctors who see more patients per week may be more concerned about their income and less by costs incurred by society. However, the relationship was similar among salaried hospital doctors, who have no such financial incentive. It is possible that hospital doctors who see fewer patients may have academic or administrative roles that cause them to be more concerned about health care costs.

### Stress from uncertainty

The association between cost-consciousness and higher tolerance for uncertainty is compatible with a previous report which linked higher anxiety due to uncertainty with higher health care expenditures in a Medicare health maintenance organisation [16]. Thus there is evidence that higher stress from uncertainty is associated with higher expenditures and, as we have found, lower cost-consciousness. From this, it follows that lower cost-consciousness and higher expenditures could be associated. To our knowledge, no study has yet studied this relationship.

### Work-related satisfaction

The association with work-related satisfaction was substantial. This is of interest as work-related satisfaction correlates with several health outcomes, in particular with the quality of care [36,37]. Although this association may be causal in either direction, we believe that work-related satisfaction may influence positively cost-consciousness: doctors who are happy about their work may think more about societal issues than unhappy doctors. Unlike other correlates of cost-consciousness, keeping doctors satisfied with their work is a modifiable factor. Nevertheless, whether improvements in doctor satisfaction translate into greater cost-consciousness remains to be seen.

### Strengths and limitations

The main strength of this study is its population-based sampling frame; the results are fairly representative of the attitudes of physicians in Geneva, Switzerland. The response rate of 59% raises the issue of selection bias, but is comparable to other surveys that depend on the cooperation of busy practitioners [2,38,39]. We could not assess response bias in depth, because the two medical organisations gave us only the adresses of their members, with no other socio-demographic information. The only caracteristic that we could track was sex, and its distribution was similar among those who responded and those who did not.

The main limitation of the study was its cross-sectional design, which precludes any formal conclusion about the causality of the associations between cost-consciousness and its correlates. Second, we were not able to obtain direct measures of doctor "costliness", such as the mean cost per patient, and thus cannot establish whether cost-consciousness influences actual costs of patient care. Third, we do not know to what extent respondents gave socially desirable answers about this rather sensitive topic. Finally, as the scales we used are not perfectly reliable, random misclassification may have caused the relationships we observed to appear weaker than they are in reality.

## Conclusion

In this study conducted in a setting where health care expenditures are among the highest in the world, doctors appeared to be generally concerned about the need to contain costs. Therefore failure to control costs does not seem to stem from a general lack of concern about costs among doctors. Nevertheless, levels of cost-consciousness were variable. Doctors in private practice, who saw the most patients, who were the least tolerant with uncertainty, and the least satisfied with their work were also the least cost-conscious.

## Competing interests

All three authors have no conflict of interest. Financial support for this study was provided entirely by a grant from the Swiss National Science Foundation (3200-053377). The funding agreement ensured the authors' independence in designing the study, interpreting the data, writing, and publishing the report.

## Authors' contributions

PAB participated in the design of the study, collected the data, performed the statistical analysis and wrote the initial manuscript. DPM participated in the design of the study and reviewed the manuscript. TVP participated in the design of the study, analysis of the data and reviewed the manuscript.

## Pre-publication history

The pre-publication history for this paper can be accessed here:


